# Cardiac strain is lower among women with HIV in relation to monocyte activation

**DOI:** 10.1371/journal.pone.0279913

**Published:** 2022-12-30

**Authors:** Mabel Toribio, Magid Awadalla, Zsofia D. Drobni, Thiago Quinaglia, Melissa Wang, Claudia G. Durbin, David A. Alagpulinsa, Lindsay T. Fourman, Giselle Alexandra Suero-Abreu, Michael D. Nelson, Takara L. Stanley, Christopher T. Longenecker, Tricia H. Burdo, Tomas G. Neilan, Markella V. Zanni

**Affiliations:** 1 Division of Endocrinology, Metabolism Unit, Massachusetts General Hospital and Harvard Medical School, Boston, MA, United States of America; 2 Department of Radiology and Division of Cardiology, Cardiovascular Imaging Research Center (CIRC), Massachusetts General Hospital and Harvard Medical School, Boston, MA, United States of America; 3 Vaccine and Immunotherapy Center, Massachusetts General Hospital and Harvard Medical School, Boston, MA, United States of America; 4 Department of Kinesiology, Applied Physiology and Advanced Imaging Laboratory, University of Texas at Arlington, Arlington, TX, United States of America; 5 Division of Cardiology and Department of Global Health, University of Washington, Seattle, WA, United States of America; 6 Department of Microbiology, Immunology, and Inflammation, Center for Neurovirology and Gene Editing, Lewis Katz School of Medicine at Temple University, Philadelphia, PA, United States of America; University of Texas Medical Branch at Galveston, UNITED STATES

## Abstract

**Background:**

Women with HIV (WWH) face heightened risks of heart failure; however, insights on immune/inflammatory pathways potentially contributing to left ventricular (LV) systolic dysfunction among WWH remain limited.

**Setting:**

Massachusetts General Hospital, Boston, Massachusetts.

**Methods:**

Global longitudinal strain (GLS) is a sensitive measure of LV systolic function, with lower cardiac strain predicting incident heart failure and adverse heart failure outcomes. We analyzed relationships between GLS (cardiovascular magnetic resonance imaging) and monocyte activation (flow cytometry) among 20 WWH and 14 women without HIV.

**Results:**

WWH had lower GLS compared to women without HIV (WWH vs. women without HIV: 19.4±3.0 vs. 23.1±1.9%, P<0.0001). Among the whole group, HIV status was an independent predictor of lower GLS. Among WWH (but not among women without HIV), lower GLS related to a higher density of expression of HLA-DR on the surface of CD14+CD16+ monocytes (ρ = -0.45, P = 0.0475). Further, among WWH, inflammatory monocyte activation predicted lower GLS, even after controlling for CD4+ T-cell count and HIV viral load.

**Conclusions:**

Additional studies among WWH are needed to examine the role of inflammatory monocyte activation in the pathogenesis of lower GLS and to determine whether targeting this immune pathway may mitigate risks of heart failure and/or adverse heart failure outcomes.

**Trial registration:**

**Clinical trials.gov registration:** NCT02874703.

## Introduction

People with HIV (PWH) on antiretroviral therapy (ART) face a two-fold increased risk of heart failure as compared to people without HIV [1–5]. PWH also have worse heart failure outcomes, including higher rates of heart failure hospitalization and cardiovascular mortality [6, 7]. Persistent monocyte activation is thought to contribute to heightened heart failure risk and adverse heart failure outcomes among PWH [5]. In vitro and animal studies have implicated monocytes and macrophages in pathologic cardiac remodeling through pro-inflammatory and pro-fibrotic phenotypes which promote myocardial fibrosis. Myocardial fibrosis, in turn, can have downstream effects on cardiac function and ultimately can progress to heart failure [8]. Notably, a recent meta-analysis suggests that risks of HIV-associated heart failure are higher in women vs. men [1]. Among PWH, sex-specific differences in heart failure risk may relate to sex-differences in systemic immune activation/inflammation [9]. Few studies, however, have focused on elucidating relevant immune pathways contributing to heart failure risk specifically among women with HIV (WWH).

Previous studies employing cardiovascular imaging techniques to interrogate heart failure risks among predominantly male cohorts of people with vs. without HIV suggest that PWH have lower global longitudinal strain (GLS), or cardiac strain [10–12]. GLS is a more sensitive measure of cardiac systolic function than ejection fraction and a lower GLS has been shown to be a robust predictor of adverse cardiac outcomes in different populations [13–15]. The value of GLS is a simple parameter that expresses longitudinal shortening of the cardiac muscle in systole as a percentage of the baseline length in diastole [16]. GLS provides meaningful information on heart failure risk, with general-population studies demonstrating that lower GLS predicts both incident heart failure and adverse heart failure outcomes [15, 17–19]. No prior studies have investigated GLS or immune correlates specifically among women with vs. without HIV, as we set out to do here. We leveraged data from a translational physiology study which included detailed immune phenotyping and cardiovascular magnetic resonance imaging (MRI) among matched cohorts of women with and without HIV with no known cardiovascular disease (CVD) [20].

## Materials and methods

### Study design and participants

Women with vs. without HIV, age 45 to 70 years, without a history CVD or diabetes were recruited from the Greater Boston Area, as previously described [20]. WWH were group-matched based on age and body mass index (BMI) to women without HIV. Twenty WWH and 14 women without HIV underwent immune and cardiovascular phenotyping, including cardiovascular MRI imaging. Data on GLS in this cohort have not previously been published. All participants provided written informed consent. This study was approved by the Massachusetts General Brigham Institutional Review Board and is registered on clinicaltrials.gov (NCT02874703).

### Cardiovascular MRI

**Fig 1:**
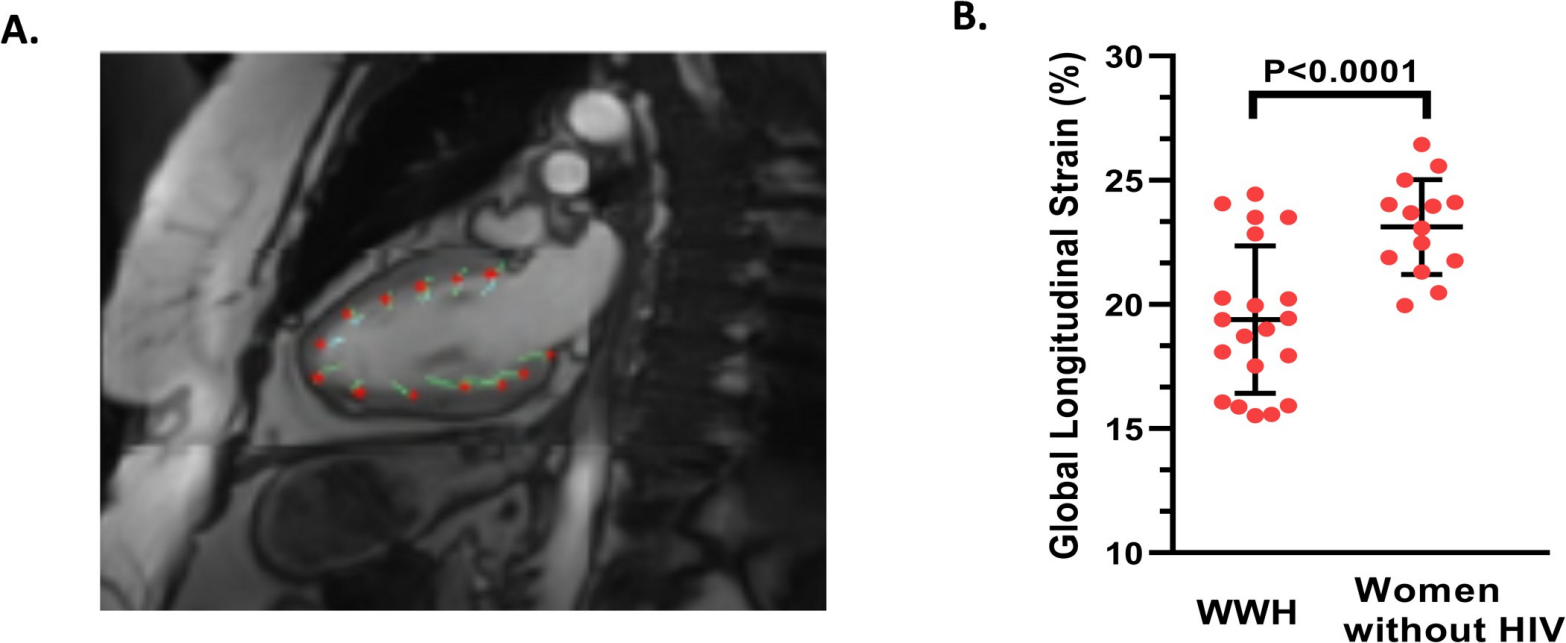
Global longitudinal strain on cardiovascular MRI among women with versus without HIV. A. Representative cardiovascular MRI image from a study participant illustrating longitudinal feature tracking of a 2- chamber cine image and analyzed using QStrain, the Medis strain software. B. Women with HIV had lower GLS compared to women without HIV. The mean and standard deviation are shown for each respective group. GLS is expressed as an absolute percentage value. Abbreviations: HIV, human immunodeficiency virus; MRI, magnetic resonance imaging; WWH, women with HIV.

The cardiovascular magnetic resonance imaging protocol was performed on a Siemens Skyra 3 Tesla MR scanner (Erlangen, Germany), as previously described [20]. Electrocardiogram (ECG)-gated b-SSFP cine sequences were acquired for long-axis 2-, 3- and 4- chamber views as well as a short axis stack. Imaging specifications included: 40 frames/cardiac cycle, pixel spacing 0.8mm x 0.8mm, 8mm slice thickness, as well as inter-slice gap, (time to echo) TE 1.5ms, (time to repetition) TR 3ms. Images were analyzed using Medis Q-strain software (Medis Medical Imaging Systems, Leiden, the Netherlands) by two readers blinded to HIV status (**Fig 1A**). GLS was characterized as follows: Feature tracking was performed in the end-diastole, and in the end-systole using Medis. The left ventricle was tracked at the endo- and epicardial borders in 2- and 4- chamber long axis views, with subsequent averaging of peak strain values to derive GLS. Tracking accuracy was visually reviewed and, if needed, corrections were made to the initial contours only. This procedure was repeated three times to determine an average GLS for each study participant. While GLS is a negative percentage, as it is calculated based on the longitudinal shortening of the myocardium during contraction, absolute percentage values are shown here to simplify data presentation. Please see **S1 File** for additional details.

### Laboratory assessments and immune parameters

Flow cytometric assessments of monocyte subpopulations were performed from cryopreserved peripheral blood mononuclear cells (PBMCs) using a BD FACsAria and analyzed using FlowJo software 8.7.1 (Treestar, Ashland, OR) [20]. The absolute number and relative percentages of monocyte subpopulations (classical, inflammatory/ intermediate and non-classical/patrolling/homing) were determined. The mean fluorescence intensity (MFI) of cell-surface receptors on various monocyte subpopulations was also determined [20]. Among WWH, CD4+ T cell counts were collected prospectively as part of their screen visit, while HIV viral load assessments were collected on the day of their imaging procedures.

### Statistical analysis

Our primary endpoint was the between-group difference in cardiac strain, quantified by the MRI-derived measure of GLS. Parameters associated with global longitudinal strain were assessed among the whole group and among sub-groups of women with vs. without HIV. Bivariate analyses were performed using either a Pearson’s or Spearman’s correlation coefficient, as appropriate. Multivariable regression modeling among the whole group was performed using global longitudinal strain as the dependent variable and atherosclerotic cardiovascular disease (ASCVD) risk score and HIV status as independent variables. Among the WWH, multivariable regression modeling was performed using GLS as the dependent variable and CD4+ T-cell count, HIV viral load, and immune parameters identified through correlational analysis as independent variables. Statistical analyses were performed using JMP Pro software (versions 16.0; SAS Institute).

## Results

### Demographic, cardiovascular, and immune parameters among women with vs. without HIV

Key demographic, cardiovascular, and immune parameters among women with vs. without HIV in this cohort have been previously published [20] and are presented in **S1 Table** for reference. Briefly, women with vs. without HIV were similar in age and BMI. WWH had evidence of diastolic dysfunction and higher left ventricular mass index, as well as elevated levels of select immune/inflammatory indices. The expression of the major histocompatibility complex (MHC) class II cell surface receptor, human-leukocyte-associated antigen-D Related (HLA-DR), on the surface of CD14+CD16+ (inflammatory) monocytes was higher among WWH compared to women without HIV [24600.5 (16933.0, 28725.8) vs. 14025.0 (11489.5, 20020.0), P = 0.001].

### Relationships between GLS and other measures of cardiac structure and function

WWH exhibited lower GLS as compared to women without HIV (GLS: 19.4±3.0 vs 23.1±1.9%, respectively, P<0.0001; **Fig 1B**). Among the whole group, after controlling for 10-year ASCVD risk score, HIV status independently predicted lower GLS (Overall Model: R^2^ = 0.38, P = 0.0008; HIV status: β-estimate = -1.98, P = 0.0002; **Table 1A**). Among the whole group, lower GLS related to lower left ventricular ejection fraction (LVEF) (r = 0.40, P = 0.02), as expected given that GLS is a measure of systolic function. Among the whole group, lower GLS was also related to lower left atrial passive EF (r = 0.38, P = 0.03) and higher left ventricular mass (ρ = -0.36, P = 0.03). Among the whole group, GLS, however, did not relate to myocardial fibrosis (r = 0.22, P = 0.23).

**Table 1:** Multivariable regression modeling for global longitudinal strain among (a) whole group and (b) women with HIV.

**a. Multivariable regression modeling for GLS among whole group**			
**Whole Model R**^**2**^ **= 0.38, P = 0.0008**			
**Covariate**	**β-estimate**	**β-SE**	**P-value**
HIV status (positive)	-1.98	0.46	**0.0002**
ASCVD Risk Score (%)	0.02	0.13	0.87
**b. Multivariable regression modeling for GLS among women with HIV**			
**Whole Model R**^**2**^ **= 0.40, P = 0.0499**			
**Covariate**	**β-estimate**	**β-SE**	**P-value**
CD4+ T-cell count (cells/mm^3^)	0.0009	0.002	0.60
HIV viral load (copies/mL)	-0.02	0.01	0.12
Expression of HLA-DR on CD14+CD16+ monocytes (MFI)	-0.0002	0.00007	**0.02**

In multivariable modeling among the whole group, HIV status remained an independent predictor of lower GLS even after controlling for ASCVD Risk Score. In multivariable modeling among women with HIV, the expression of HLA-DR on CD14+CD16+ (inflammatory) monocytes remained an independent predictor of lower GLS even after controlling for the HIV-specific parameters of CD4+ T-cell count and HIV viral load.

Abbreviations: ASCVD, atherosclerotic cardiovascular disease; β-estimate, beta-estimate; β-SE, beta-standard error; CD4, cluster of differentiation 4; CD14, cluster of differentiation 14; CD16, cluster of differentiation 16; GLS, global longitudinal strain; HIV, Human Immunodeficiency Virus; HLA-DR, human-leukocyte-associated antigen-D Related; MFI, mean fluorescence index

### Relationship between GLS and immune parameters

Among the whole group and among WWH (but not among women without HIV), lower GLS related to a higher density of expression of HLA-DR on the surface of CD14+CD16+ (inflammatory) monocytes, reflective of monocyte activation (Whole Group: ρ = -0.53, P = 0.001; WWH: ρ = -0.45, P = 0.0475). Further, among WWH, the density of expression of HLA-DR on the surface of CD14+CD16+ monocytes remained an independent predictor of lower GLS even after controlling for CD4+ T-cell count and HIV viral load (Whole Model: R^2^ = 0.40, P = 0.0499; CD14+CD16+ MFI of HLA-DR: β-estimate = -0.0002, P = 0.02; **Table 1B**). Among the whole group, lower GLS related to higher levels of soluble CD14 (ρ = -0.46, P = 0.006), but not to levels of soluble CD163 (r = 0.26, P = 0.14) and MCP-1/CCL2 (r = 0.15, P = 0.41). Among the whole group and among sub-groups, GLS did not relate to the absolute and percentage of CD14+CD16- (classical) monocytes (Absolute CD14+CD16- monocytes: Whole Group: ρ = 0.08, P = 0.68, WWH: ρ = -0.09, P = 0.70; women without HIV: ρ = 0.47, P = 0.10 and Percentage of CD14+CD16- monocytes: Whole Group: ρ = -0.24, P = 0.18, WWH: ρ = 0.02, P = 0.93; women without HIV: ρ = -0.12, P = 0.69), CD14+CD16+ (inflammatory/intermediate) monocytes (Absolute CD14+CD16+ monocytes: Whole Group: ρ = 0.11, P = 0.53, WWH: ρ = -0.06, P = 0.80; women without HIV: ρ = 0.26, P = 0.39 and Percentage of CD14+CD16+ monocytes: Whole Group: ρ = 0.17, P = 0.34, WWH: ρ = 0.08, P = 0.75; women without HIV: ρ = -0.13, P = 0.67), and CD14-CD16+ (non-classical/patrolling/homing) monocytes (Absolute CD14-CD16+ monocytes: Whole Group: ρ = 0.23, P = 0.20, WWH: ρ = -0.15, P = 0.52; women without HIV: ρ = 0.48, P = 0.10 and Percentage of CD14-CD16+ monocytes: Whole Group: ρ = 0.28, P = 0.12, WWH: ρ = 0.06, P = 0.79; women without HIV: ρ = 0.27, P = 0.36).

### Relationship between GLS and ART use among WWH

Among our cohort of WWH on ART, GLS did not relate to the duration of ART use (ρ = -0.01, P = 97).

## Discussion

In our study, WWH without known CVD had lower GLS, even after controlling for traditional CVD risk factors. Among the whole group and WWH (but not among women without HIV), the density of expression of HLA-DR on the surface of CD14+CD16+ (inflammatory) monocytes, reflective of inflammatory monocyte activation, related inversely to GLS and remained a predictor of lower GLS among WWH, even after controlling for HIV-specific factors. Together, these data suggest that among WWH, inflammatory monocyte activation – through effects on cardiac muscle function – may play a role in heightened heart failure risk.

In our study, women with (vs. without) HIV had lower GLS, which presages and predicts heart failure and adverse heart failure outcomes [18, 19]. These findings are directionally consistent with findings from a prior study among a predominantly-male cohort employing cardiovascular MRI [12]. Of note, a study of individuals with vs. without HIV in Uganda employing speckle-tracking echocardiography also revealed lower GLS among PWH, 60% of whom were women [21]. In our cohort of United States (U.S.) women, HIV infection predicted lower GLS even after controlling for ASCVD risk score, which encompasses age, sex, race, blood pressure, lipid levels, smoking status, history of diabetes, and current anti-hypertensive treatment. This finding suggests a potential pathway – beyond traditional CVD risk factors – through which HIV may augment risks of heart failure and adverse heart failure outcomes in this vulnerable population.

Higher density of expression of HLA-DR on the surface of CD14+CD16+ (inflammatory) monocytes remained a strong predictor of lower GLS among WWH in our study cohort, even after controlling for HIV-specific parameters. Inflammatory monocytes have a pro-inflammatory cytokine signature [22] and highly express the MHC class II receptor HLA-DR on their surface [23]. A higher density of expression of HLA-DR on the surface of inflammatory monocytes reflects a greater degree of monocyte activation. A prior U.S. study employing cardiovascular MRI to interrogate heart failure risk among a predominately male cohort of individuals with vs. without HIV revealed that lower radial strain (another cardiovascular MRI-derived measure of cardiac systolic function) was associated with higher systemic levels of the marker of monocyte activation, MCP-1, specifically among the sub-group with HIV [11]. While this study supports our own findings, it also differs in several respects including the measure of cardiac strain used (radial strain vs. longitudinal strain) and the study population (predominately male cohort of PWH vs. WWH). Future studies on sex-specific relationships between immune activation and lower GLS among PWH across regions are needed.

Evaluating sex-specific immune mechanisms of CVD risk among WWH is crucial given sex-differences in immune responses to HIV infection and sex-differences in relationships between immune indices and CVD risk surrogates among PWH. In response to HIV infection, women demonstrate higher levels of toll-like receptor signaling [9] and T-cell activation [24]. Sex-differences in the immune response to HIV infection may contribute to heightened levels of immune/inflammatory markers among WWH. In a recent study of ART-treated PWH in the U.S., WWH demonstrated higher levels of systemic markers of immune activation compared to men with HIV, even after controlling for ASCVD risk [25]. Further, select immune parameters associated significantly with CVD risk surrogates among WWH but not among men with HIV [25]. Conversely, in our current study, we did not find higher levels of inflammatory monocytes among WWH on ART compared to women without HIV, while among predominately male cohort of PWH on ART in separate study recruited from the same geographic region and similar entry criteria, we did find higher levels of inflammatory monocytes among PWH on ART [26]. To elucidate immune mechanisms contributing to heightened CVD risk specifically among WWH, we need more studies which either focus exclusively on WWH or which robustly enroll WWH, thereby enabling the performance of appropriately powered sex-stratified analyses.

Our study was limited by the cross-sectional design, which precludes inferences on causality. The participants in our study were recruited from a U.S. metropolitan area, which may limit the generalizability of our results to other regions. Our study was also limited by its relatively small sample size and the potential influence of unmeasured confounders on the between-group differences on GLS. A key strength of our study was the focus on WWH, a population underrepresented in CVD risk research. Further, our data were prospectively collected using cardiovascular MRI and detailed metabolic/immune phenotyping procedures. Lastly, our participants reflected the racial/ethnic diversity of the demographic of WWH in the Greater Boston area.

## Conclusion

In our study, we applied cardiovascular MRI to quantify GLS in matched cohorts of US women with vs. without HIV. WWH (compared to age- and BMI- group matched women without HIV) had lower GLS; and HIV status was an independent predictor of lower GLS, controlling for traditional CVD risk. Inflammatory monocyte activation, in turn, related to lower GLS among WWH but not among women without HIV. Future studies are needed to determine if inflammatory monocyte activation (independent of CD4+T-cell count and HIV viral load) may contribute etiologically to lower cardiac strain among WWH on ART. Further, while in our study duration of ART use did not relate to lower GLS, future studies exploring the potential role of ART on inflammation and downstream adverse cardiac modeling among WWH are needed. Additional studies are also needed to investigate whether targeting inflammatory monocyte activation among WWH may prevent progression of subclinical LV systolic dysfunction to heart failure and adverse heart failure outcomes in this at-risk population.

## Supporting information

S1 Table Baseline characteristics among women with and without HIV. Normally distributed variables are presented as mean ± standard deviation (SD); non-normally distributed data are presented as median (interquartile range; IQR). P-values were determined by student’s two-tailed *t*-test, Wilcoxon rank-sum test, and chi-square test for normally distributed, non-normally distributed, and categorical variables, respectively. There were no significant differences in baseline characteristics among women with versus without HIV. Select systemic markers of monocyte activation (MCP-1, sCD14, and sCD163) were higher among women with HIV versus without HIV. The expression of HLA-DR on the surface of CD14+CD16+ (inflammatory) monocytes, reflective of inflammatory monocyte activation, was higher among women with versus without HIV. *Lower limit of detection for the HIV viral load assay employed was 20 copies/mL. Values of 19 copies/mL were imputed when viral load was undetectable. **Expression of HLA-DR on monocyte subpopulations could not be obtained for one woman without HIV. Abbreviations: ART, anti-retroviral therapy; ASCVD, atherosclerotic cardiovascular disease; BMI, body mass index; CCR2, C-C chemokine receptor type 2; CCR5, C-C chemokine receptor type 5; CD4, cluster of differentiation 4; CD14, cluster of differentiation 14; CD16, cluster of differentiation 16; CXCL10, C-X-C motif chemokine 10; HDL-C, high-density lipoprotein cholesterol; HCV, hepatitis C virus; HIV, Human Immunodeficiency Virus; HLA-DR, human-leukocyte-associated antigen-D Related; INSTI, integrase inhibitor; LDL-C, low-density lipoprotein cholesterol; LMP, last menstrual period; MCP-1, monocyte chemoattractant protein 1; MFI, mean fluorescence intensity; NNRTIs, non-nucleoside reverse transcriptase inhibitors; NRTIs, nucleoside reverse transcriptase inhibitors; PBMCs, peripheral blood mononuclear cells; PIs, protease inhibitors; sCD14, soluble CD14; sCD163, soluble CD163; WHIV, women with HIV; WHR, waist to hip ratio.
(DOCX)

S1 File Global longitudinal strain supplemental methods. Additional details on the assessment of global longitudinal strain using cardiovascular MRI can be found in our Supplemental Methods.
(DOCX)
