# Supplementary material for: Cardiac strain is lower among women with HIV in relation to monocyte activation
Source: PLoS One. 2022 Dec 30;17(12):e0279913. doi: 10.1371/journal.pone.0279913 (PMC9803182; doi:10.1371/journal.pone.0279913)
Supplement: S1 Table — Normally distributed variables are presented as mean ± standard deviation (SD); non-normally distributed data are presented as median (interquartile range; IQR). P-values were determined by student’s two-tailed t-test, Wilcoxon rank-sum test, and chi-square test for normally distributed, non-normally distributed, and categorical variables, respectively. There were no significant differences in baseline characteristics among women with versus without HIV. Select systemic markers of monocyte activation (MCP-1, sCD14, and sCD163) were higher among women with HIV versus without HIV. The expression of HLA-DR on the surface of CD14+CD16+ (inflammatory) monocytes, reflective of inflammatory monocyte activation, was higher among women with versus without HIV. *Lower limit of detection for the HIV viral load assay employed was 20 copies/mL. Values of 19 copies/mL were imputed when viral load was undetectable. **Expression of HLA-DR on monocyte subpopulations could not be obtained for one woman without HIV. Abbreviations: ART, anti-retroviral therapy; ASCVD, atherosclerotic cardiovascular disease; BMI, body mass index; CCR2, C-C chemokine receptor type 2; CCR5, C-C chemokine receptor type 5; CD4, cluster of differentiation 4; CD14, cluster of differentiation 14; CD16, cluster of differentiation 16; CXCL10, C-X-C motif chemokine 10; HDL-C, high-density lipoprotein cholesterol; HCV, hepatitis C virus; HIV, Human Immunodeficiency Virus; HLA-DR, human-leukocyte-associated antigen-D Related; INSTI, integrase inhibitor; LDL-C, low-density lipoprotein cholesterol; LMP, last menstrual period; MCP-1, monocyte chemoattractant protein 1; MFI, mean fluorescence intensity; NNRTIs, non-nucleoside reverse transcriptase inhibitors; NRTIs, nucleoside reverse transcriptase inhibitors; PBMCs, peripheral blood mononuclear cells; PIs, protease inhibitors; sCD14, soluble CD14; sCD163, soluble CD163; WHIV, women with HIV; WHR, waist to hip ratio. (DOCX) [file pone.0279913.s001.docx]

**Supplemental Table 1**: **Baseline Characteristics Among Women with and without HIV**

|  | **Women with HIV** (n=20) | **Women without HIV** (n=14) | **P-value** |
| --- | --- | --- | --- |
| **Demographic and Traditional Cardiovascular Disease Risk Parameters** | | | |
| Age, years | 52 ± 4 | 53 ± 6 | 0.61 |
| Race, % |  |  | 0.32 |
| White | 40 (8/20) | 64 (9/14) |  |
| Black/African American | 40 (8/20) | 29 (4/14) |  |
| Other | 20 (4/20) | 7 (1/14) |  |
| Ethnicity, Hispanic, % | 10 (2/20) | 14 (2/14) | 0.70 |
| Current hypertension, % | 25 (5/20) | 29 (4/14) | 0.82 |
| Current antihypertensive, % | 15 (3/20) | 7 (1/14) | 0.47 |
| Current smoking, % | 50 (10/20) | 29 (4/14) | 0.21 |
| Current cocaine use, % | 0 (0/20) | 0 (0/14) | -- |
| Total cholesterol, mg/dL | 200 ± 41 | 203 ± 28 | 0.80 |
| LDL-C, mg/dL | 113 ± 33 | 109 ± 26 | 0.69 |
| HDL-C, mg/dL | 57 ± 15 | 67 ± 24 | 0.23 |
| Triglycerides, mg/dL | 119 (91, 175) | 92 (60, 176) | 0.23 |
| 10-year ASCVD risk score, % | 3.6 (1.3, 4.8) | 2.1 (1.3, 6.8) | 0.67 |
| Hemoglobin A1c, % | 5.6 ± 0.3 | 5.5 ± 0.3 | 0.50 |
| BMI, kg/m^2^ | 32 ± 7 | 32 ± 7 | 0.73 |
| WHR | 0.9 ± 0.1 | 0.9 ± 0.1 | 0.36 |
| Current HCV, % | 10 (2/20) | 0 (0/14) | 0.14 |
| **HIV-specific Parameters** | | | |
| Time since HIV diagnosis, years | 19 ± 8 | -- | -- |
| Total duration ART, years | 18 (8, 22) | -- | -- |
| NRTI use, % | 80 (16/20) | -- | -- |
| NNRTI use, % | 30 (6/20) | -- | -- |
| PI use, % | 45 (9/20) | -- | -- |
| INSTI use, % | 65 (13/20) | -- | -- |
| CCR5 antagonist use, % | 5 (1/20) | -- | -- |
| CD4^+^ T cell count, cells/mm^3^ | 773 (526, 1202) | -- | -- |
| Nadir CD4^+^ T cell count, cells/mm^3^ | 138 (18, 270) | -- | -- |
| HIV viral load undetectable, % | 84 (16/19) | -- | -- |
| HIV viral load, copies/mL* | 19 (19, 19) | -- | -- |
| **Hormonal Status** | | | |
| Time since LMP, years | 6 (2, 10) | 5 (0, 13) | 0.79 |
| Current hormone therapy, % | 0 (0/20) | 0 (0/14) | -- |
| **Cardiac Parameters** | | | |
| Left ventricular mass index (g/m^2^) | 48.4 (43.9, 56.7) | 42.3 (40.4, 47.2) | **0.01** |
| Left ventricular ejection fraction (%) | 58 ± 4 | 60 ± 5 | 0.19 |
| Left atrial passive ejection fraction (%) | 26.9 ± 9.5 | 35.0 ± 6.8 | **0.007** |
| **Circulating Systematic Immune Biomarkers** | | | |
| MCP-1 pg/mL | 210 ± 43 | 167 ± 45 | **0.009** |
| CXCL10, pg/mL | 152 (100, 206) | 107 (97, 135) | 0.10 |
| sCD14, ng/mL | 1904 (1551, 2224) | 1501 (1329, 1668) | **0.008** |
| sCD163, ng/mL | 1260 ± 293 | 938 ± 308 | **0.005** |
| **Circulating Monocyte Subpopulations** | | | |
| % of monocytes from total PBMCs | 8.2 ± 4.1 | 6.8 ± 4.7 | 0.40 |
| % CD14+CD16- (% of total monocytes) | 84.7 (81.8, 88.8) | 81.2 (72.7, 87.4) | 0.20 |
| Absolute number of CD14+CD16- cells per µL | 347 (229, 420) | 364 (301, 476) | 0.41 |
| % CD14+CD16+ (% of total monocytes) | 7.5 (6.8, 10.0) | 10.8 (6.2, 12.6) | 0.22 |
| Absolute number of CD14+CD16+ cells per µL | 31 (23, 43) | 33 (25, 68) | 0.27 |
| % CD14-CD16+ (% of total monocytes) | 5.9 (3.5, 9.1) | 6.7 (4.7, 11.9) | 0.26 |
| Absolute number of CD14-CD16+ cells per µL | 23 (16, 36) | 27 (17, 81) | 0.22 |
| **Cellular Expression Profile of Circulating Monocyte Subpopulations**** | | | |
| Expression of HLA-DR on CD14+CD16- monocytes (MFI) | 6219.0 (5470.5, 8813.8) | 6015.0 (4666.5, 6690.5) | 0.17 |
| Expression of HLA-DR on CD14+CD16+ monocytes (MFI) | 24600.5 (16933.0, 28725.8) | 14025.0 (11489.5, 20020.0) | **0.001** |
| Expression of HLA-DR on CD14-CD16+ monocytes (MFI) | 10772.5 (7644.8, 13425.0) | 10284.0 (8835.5, 11356.0) | 0.54 |

Normally distributed variables are presented as mean ± standard deviation (SD); non-normally distributed data are presented as median (interquartile range; IQR). P-values were determined by student’s two-tailed *t*-test, Wilcoxon rank-sum test, and chi-square test for normally distributed, non-normally distributed, and categorical variables, respectively.

There were no significant differences in baseline characteristics among women with versus without HIV. Select systemic markers of monocyte activation (MCP-1, sCD14, and sCD163) were higher among women with HIV versus without HIV. The expression of HLA-DR on the surface of CD14+CD16+ (inflammatory) monocytes, reflective of inflammatory monocyte activation, was higher among women with versus without HIV.

*Lower limit of detection for the HIV viral load assay employed was 20 copies/mL. Values of 19 copies/mL were imputed when viral load was undetectable.

**Expression of HLA-DR on monocyte subpopulations could not be obtained for one woman without HIV

Abbreviations: ART, anti-retroviral therapy; ASCVD, atherosclerotic cardiovascular disease; BMI, body mass index; CCR2, C-C chemokine receptor type 2; CCR5, C-C chemokine receptor type 5; CD4, cluster of differentiation 4; CD14, cluster of differentiation 14; CD16, cluster of differentiation 16; CXCL10, C-X-C motif chemokine 10; HDL-C, high-density lipoprotein cholesterol; HCV, hepatitis C virus; HIV, Human Immunodeficiency Virus; HLA-DR, human-leukocyte-associated antigen-D Related; INSTI, integrase inhibitor; LDL-C, low-density lipoprotein cholesterol; LMP, last menstrual period; MCP-1, monocyte chemoattractant protein 1; MFI, mean fluorescence intensity; NNRTIs, non-nucleoside reverse transcriptase inhibitors; NRTIs, nucleoside reverse transcriptase inhibitors; PBMCs, peripheral blood mononuclear cells; PIs, protease inhibitors; sCD14, soluble CD14; sCD163, soluble CD163; WHIV, women with HIV; WHR, waist to hip ratio
