# Supplementary material for: Cardiac strain is lower among women with HIV in relation to monocyte activation
Source: PLoS One. 2022 Dec 30;17(12):e0279913. doi: 10.1371/journal.pone.0279913 (PMC9803182; doi:10.1371/journal.pone.0279913)
Supplement: S1 File — Additional details on the assessment of global longitudinal strain using cardiovascular MRI can be found in our Supplemental Methods. (DOCX) [file pone.0279913.s002.docx]

**Supplemental Methods**

Left ventricular ejection fraction (LVEF) is a global measure of all the myocardial layers and serves as an indirect measure of LV contractility and function. However, while LVEF is the result of the sum of regional functions, the individual contributions of these regions cannot be determined through LVEF alone. The three layers of the left ventricular wall include the subendocardial, mid-wall, and subepicardial layers, each with fibers oriented in a particular direction resulting in deformation along three individual axes. The subendocardial and subepicardial layers have fibers oriented longitudinally from the base to the apex while the mid-wall layer has circumferentially oriented fibers. Global Longitudinal Strain (GLS) characterizes the shortening of the myocardium from the base to the apex along the long-axis. Studies have shown normal values using feature-tracking cardiac magnetic resonance (CMR-FT) in healthy individuals with a GLS value of -21.3 ± 4.8%.[1] CMR techniques for cardiac strain include imaging techniques like displacement encoding with stimulated echoes (DENSE), strain encoding (SENC), Spatial Modulation of Magnetization (SPAMM), and image analysis techniques like Harmonic Phase (HARP) and feature-tracking (FT) which is what we used. This tissue tracking/feature tracking software used by Medis QSTRAIN employs an optical flow method, in which recognition of patterns of features can be tracked throughout the cardiac cycle. The tracking begins by identifying a small region of interest (ROI) in one image and searching for the most comparable gray-scale pattern in the subsequent phase.

**Reference**

1. Morton G, Schuster A, Jogiya R, Kutty S, Beerbaum P, Nagel E. Inter-study reproducibility of cardiovascular magnetic resonance myocardial feature tracking. J Cardiovasc Magn Reson. 2012;14:43. Epub 2012/06/23. doi: 10.1186/1532-429X-14-43. PubMed PMID: 22721175; PubMed Central PMCID: PMCPMC3461471.
